# Intracranial hypertension due to bilateral internal jugular venous occlusion in eagle syndrome: a case report

**DOI:** 10.1016/j.heliyon.2023.e16188

**Published:** 2023-05-11

**Authors:** Lu Xiong, Zigao Wang

**Affiliations:** a Department of Anesthesiology, Tinglin Hospital, Jinshan District, Shanghai, China; b Department of Neurology, Huashan Hospital, Fudan University, Shanghai, China; c Department of Neurology, Kashi Prefecture Second People's Hospital, XinJiang, China

**Keywords:** Intracranial hypertension, Eagle syndrome, Jugular venous, Styloid process

## Abstract

Eagle syndrome is a clinical condition that characterized by myriad of symptoms associated with the compression of neurovascular structures by an elongated styloid process. Herein we describe a rare case of Eagle syndrome who showed bilateral internal jugular venous occlusion duo the compression of the styloid process. A young man presented with headaches for six months. Lumbar puncture showed an opening pressure of 260 mmH_2_O and the cerebrospinal fluid analysis was normal. Catheter angiography revealed occlusion of bilateral jugular venous. Computed tomography venography demonstrated compression of bilateral jugular venous by bilateral elongated styloid processes. The patient was diagnosed with Eagle syndrome and suggested to undergo styloidectomy, after which he recovered completely. We emphasize that Eagle syndrome is a rare cause of intracranial hypertension and styloid resection can bring an excellent clinical outcome in patents with intracranial hypertension due to Eagle syndrome.

## 1 Introduction

Eagle syndrome is a rare clinical condition that characterized by myriad of symptoms associated with the compression of neurovascular structures by an elongated styloid process, including oropharyngeal pain, dysphagia, and a foreign body sensation [1,2]. However, cases presenting with intracranial hypertension in Eagle syndrome have rarely been reported. Herein we described a unique case of Eagle syndrome presenting with intracranial hypertension which resulted from the external compression and subsequent occlusion of bilateral jugular venous by the elongated styloid processes.

## 2 Case presentation

**Fig. 1:**
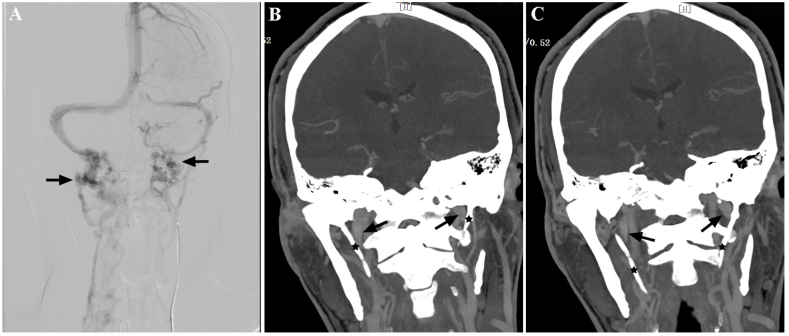
(A) Catheter angiography shows bilateral occlusion of internal jugular venous (arrows) and adjacent collaterals. (B–C) Computed tomography venography shows bilateral elongated styloid processes (asterisks) and compression of adjacent internal jugular venous (arrows).

A previously healthy 36-year-old man presented with constant non-throbbing headaches for six months. The headaches worsened when lying and improved when standing up. He experienced several episodes of blurred vision and tinnitus. Physical examination demonstrated normal body mass index, normal neurological examination, and normal visual acuity. Fundoscopy was unremarkable and his visual fields were full. Lumbar puncture and cerebrospinal fluid analysis were negative except an opening pressure of 260 mmH_2_O. Brain magnetic resonance image was normal except empty sella turcica. Idiopathic intracranial hypertension was suspected. Therefore, transfemoral selective catheter cerebral angiography was performed to explore the underlying sinus stenosis. However, no evidence of sinus stenosis was found. Interestingly, occlusion of bilateral jugular venous at C1 vertebral level was revealed (Fig. 1A). Further, cranial and cervical computed tomography venography confirmed the jugular venous occlusion and demonstrated bilaterally elongated styloid processes measured 6.2 cm on the right side and 5.0 cm on the left side (Fig. 1B and C). The patient was finally diagnosed with intracranial hypertension due to Eagle syndrome. He was transferred to an ear-nose-throat hospital where he was performed with right-sided styloidecotmy and C1 tuberculectomy via transcervical approach, after which his headache recovered completely. He refused repeated neuroimage screening and lumbar puncture because he was symptom free after surgical removal of the elongated styloid process even without taking any medications.

## 3 Discussion

Eagle syndrome, first reported in 1937, is a rare and poorly understood clinical entity that results from the compression of neurovascular structures by an abnormal styloid process which is longer than 3.0 cm [1,3]. It can be divided into three subtypes based on the different structures compressed by the elongated styloid process [4]. Type one classically presents with neck pain, dysphagia, foreign body sensation, and otalgia associated with compression of cranial nerves V, VII, IX, or X. Type two typically presents with transient ischemic stroke or stroke related to the compression of internal carotid artery. Type three often presents with intracranial hypertension resulting from the compression of jugular venous and subsequent intracranial venous outflow obstruction.

Since its first report in 2011, more than 30 cases of Eagle syndrome with jugular venous compression have been reported [4]. Jugular venous compression by elongated styloid process will become symptomatic in the context of bilateral styloid compression, dominant venous system compression, or contralateral venous occlusion. The clinical features in Eagle syndrome with jugular venous compression mainly compromise headaches, vomiting, and papilloedema which is similar to what often happens in cases with idiopathic intracranial hypertension (IIH). A previous study showed that patients with styloidogenic jugular venous compression had lower rate of obesity and higher rate of positional headache compared with those with IIH. Moreover, they had a longer styloid process and a shorter distance between the styloid process and the C1 lateral tubercle [5]. These distinct clinical characteristics will help to distinguish styloidogenic jugular venous compression from IIH.

Management of Eagle syndrome comprised conservative medication and more definitive surgical treatment [1]. Local pain associated with eagle syndrome can be treated with a combination of analgesics, anticonvulsants, antidepressants, and local injections. However, surgical treatment offers long-lasting symptomatic relief and a definitive treatment especially in patients with compression of the adjacent internal carotid artery and jugular venous [4,6]. Both intraoral and transcervical approach of styloidectomy have been demonstrated to be effective in removing the elongated styloid process and subsequently result in symptom relief in patients with Eagle syndrome. Although transcervical approach carries disadvantages of a scar and risk of injury to the facial nerve, it is associated with shorter operation time, lower rate of postoperative infections, and complete exposure of the styloid process [1,6]. As to the jugular vein compression in Eagle syndrome, it has been suggested that C1 tuberculectomy is an essential and unique technique to relive the compression of jugular vein between the styloid process and the C1 tubercle [5]. Therefore, transcervical approach is preferred for Eagle syndrome with jugular vein compression.

## 4 Conclusion

Our present case highlights that Eagle syndrome is a rare cause of intracranial hypertension and evaluation of the jugular vein and adjacent styloid process length is mandatory for patients suspected of intracranial hypertension. Styloid resection can bring an excellent clinical outcome in patents with intracranial hypertension due to Eagle syndrome.

## Author contribution statement

All authors listed have significantly contributed to the development and the writing of this article.

## Data availability statement

Data will be made available on request.

## Funding

None.

## Declaration of competing interest

The authors declared no conflicts of interest.
